# The Impact of Orthodontic Treatment on Masseter Muscle Development in Pediatric Patients: A One-Year Follow-Up Study

**DOI:** 10.3390/jcm14228175

**Published:** 2025-11-18

**Authors:** Stavros Kiliaridis, Aikaterini Frasiola, Ioanna Georgiakaki, Maria Charalampidou, Gregory S. Antonarakis

**Affiliations:** 1 Division of Orthodontics, University Clinics of Dental Medicine, University of Geneva, 1205 Geneva, Switzerland; stavros.kiliaridis@unige.ch (S.K.); maria.charalampidou@etu.unige.ch (M.C.); gregory.antonarakis@unige.ch (G.S.A.); 2 Department of Orthodontics and Dentofacial Orthopedics, University of Bern, 3011 Bern, Switzerland; 3 Independent Researcher, 52070 Aachen, Germany; ioanna.georgiakaki@gmail.com

**Keywords:** diagnostic imaging, masseter muscle thickness, ultrasonography, orthodontic appliances

## Abstract

**Background/Objectives:** This study aimed to evaluate the functional adaptation of the masseter muscle in growing individuals after one year of orthodontic treatment by assessing changes in its thickness. **Methods:** Twenty children with a mean age of 10.4 ± 2.1 years undergoing orthodontic treatment were monitored over one year. Ultrasonographic measurements of masseter muscle thickness were taken before the commencement of orthodontic treatment and one year later. Eighteen orthodontically untreated children with a mean age of 9.9 ± 2.0 years served as the control group; their masseter muscle thickness was measured at baseline and after the same follow-up period. Comparisons were made between the two groups. **Results:** At baseline, the mean masseter muscle thickness was 11.4 ± 1.3 mm in the control group and 11.7 ± 1.4 mm in the treatment group. After one year, children in the untreated control group showed an average increase of 0.5 ± 0.6 mm (*p* < 0.001) in the thickness of the masseter muscle, whereas those undergoing orthodontic treatment exhibited an average decrease of 0.6 ± 0.7 mm (*p* < 0.001). Multiple regression analysis accounting for age, gender, and initial masseter muscle thickness indicated that orthodontic treatment resulted in a reduction in masseter muscle thickness by 1.1 mm (*p* < 0.001) compared with the untreated control group. **Conclusions:** Orthodontic treatment may influence the development of the masticatory muscles. In our sample, the masseter muscle showed an estimated atrophy of approximately 9% after one year of orthodontic treatment compared with the thickness it would likely have achieved in the absence of any orthodontic intervention.

## 1. Introduction

Orthodontic treatment in children and adolescents is commonly undertaken to improve esthetic and functional problems associated with malocclusions. During active orthodontic treatment, tooth movement can significantly influence orofacial function, such as masticatory activity [1,2]. The orthodontic appliances used along with the changes in the occlusal contacts that occur, especially during the initial phase of treatment [3,4], may affect the subjects’ central nervous system (CNS). Previous studies have demonstrated that both functional appliances and orthodontic fixed appliances can alter masticatory muscle activity [5,6,7,8]. These alterations in muscle activity, as manifested by a decrease in bite force and electromyographic muscle activity, are thought to result from the temporary pain and discomfort associated with orthodontic forces [9,10,11].

The neurophysiological basis of these motor adjustments can be interpreted by the pain adaptation model. According to this paradigm, nociceptive stimulation induces a protective neuromuscular response characterized by the reversible suppression of agonist muscle activity and modest facilitation of antagonist muscles. This mechanism serves to limit mechanical loading and results in a controlled reduction in movement velocity and bite force [9,12,13].

Complementary evidence suggests that orthodontic interventions may induce neuroplastic changes in the corticomotor control of the masticatory muscles, reflecting an adaptive response of the CNS to the altered oral environment [14]. Given the potential influence of orthodontic treatment on the masticatory muscles, it is reasonable to hypothesize that their development may be affected by such interventions, leading to a different growth pattern in individuals undergoing orthodontic treatment compared with those who remain orthodontically untreated.

A reliable method for evaluating muscle functional profile involves measurements of its thickness or cross-sectional area. Measurements obtained from computed tomography have been shown to correlate well with the anatomic cross-sections of dissected masticatory muscles [15]. Moreover, the masseter muscle has been identified as a good representative muscle of all the masticatory elevator muscles, with a strong association between its size and the size of the other elevator muscles [16]. Because of the significant disadvantage of exposing patients to ionizing radiation, ultrasonography has become a preferred non-invasive method for assessing masseter muscle thickness [17]. The validity of ultrasonographic measurements has been confirmed by studies demonstrating strong agreement between ultrasonographic and magnetic resonance imaging (MRI) measurements of the masseter muscle [18].

The aim of the present study was to evaluate whether masticatory muscle capacity, represented by the thickness of the masseter muscle, is affected by orthodontic treatment over a one-year period. The null hypothesis was that no significant difference would be found in masseter muscle thickness between orthodontically treated and untreated growing children after one year of observation.

## 2. Materials and Methods

### 2.1. Ethical Considerations

The present study was approved by the local research ethics board (protocol number 00195; approval date: 3 July 2024), and informed consent was received from all participants and their parents or guardians.

### 2.2. Study Design and Participants

This retrospective cohort study included a total of 38 growing children, divided into an orthodontically treated group and a control group consisting of subjects without immediate need of orthodontic treatment. All of the included subjects were patients in a single-centre orthodontic office, treated between 1997 and 1999. For each subject two ultrasonographic recordings of the masseter muscles were available, namely an initial pre-treatment recording and a second recording obtained one year later (either one year into orthodontic treatment or after a period of one year without treatment). In the orthodontically treated group, the initial recording was performed within six weeks prior the insertion of orthodontic appliances. All subjects in the treatment group were still undergoing active orthodontic treatment at the time of the second recording.

The inclusion criteria for the treatment group comprised growing, healthy children aged 7–15 years, who required orthodontic treatment and demonstrated satisfactory compliance throughout therapy. Exclusion criteria comprised the presence of temporomandibular disorders, systemic or neuromuscular diseases, craniofacial syndromes or facial clefts, and a history of prior orthodontic treatment. Identical inclusion and exclusion criteria were applied to the control group to ensure comparability between groups, with the exception of the inclusion criterion of requiring orthodontic treatment and demonstrating satisfactory compliance throughout therapy, as they were without immediate need for orthodontic treatment.

The treatment group consisted of 20 children (13 females, 7 males), with a mean age of 10.4 ± 2.1 years at the start of treatment. The first ultrasonographic recording of the masseter muscle was obtained just before the start of active orthodontic treatment (T0), while the second recording was undertaken approximately 1 year later (T1; mean follow up during treatment 14.2 ± 2.6 months).

The control group consisted of 18 children (13 females, 5 males), with a mean age of 9.9 ± 2.0 years. These children came to the orthodontic office for an initial consultation, but did not require immediate orthodontic treatment. As part of the consultation, an ultrasonographic assessment of masseter muscle thickness was performed and repeated approximately one year later (mean follow up period 13.2 ± 2.0 months).

### 2.3. Sample Size Calculation

The sample size of the present study was calculated using G*Power analysis software version 3.1.9.7 [19], with an alpha level of 0.05 and a beta of 0.85 for a two-tailed *t*-test. The expected effect size was estimated from a previous study by Kiliaridis et al. [20], which compared changes in masseter muscle thickness over a one-year period between orthodontically treated and untreated individuals. Based on their data, a Cohen’s d effect size of 1.88 was calculated, indicating that a total of 28 subjects would be sufficient to achieve adequate power, i.e., 14 subjects in each group. In order to ensure subgroup representation and accommodate potential variability, the sample size was conservatively increased, resulting in a final sample of 38 participants (20 treated and 18 controls).

### 2.4. Ultrasonographic Thickness of Masseter Muscle

Masseter muscle thickness was measured using ultrasonography. A single examiner (IG) calibrated to the senior author (SK) conducted all the ultrasonographic recordings both in the treatment and the control groups, using the method described by Kiliaridis and Kälebo [17] and later modified by Raadsheer et al. [18] All ultrasonographic recordings were conducted with a real-time scanner (Scanner 480, Pie Medical, Maastricht, The Netherlands) with a 7.5 MHz linear array transducer.

**Figure 1:**
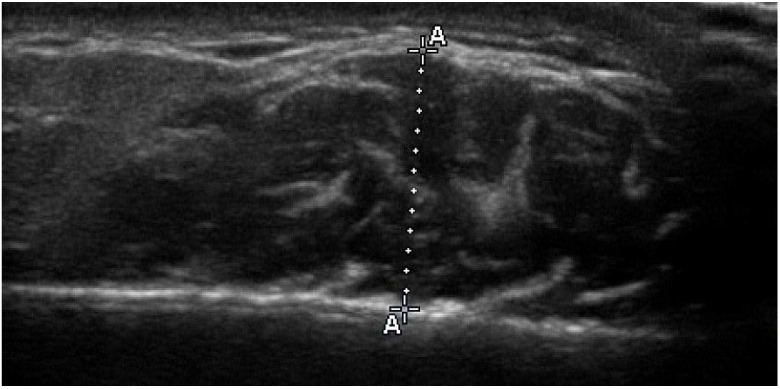
Transverse ultrasound image of the masseter muscle during contraction. The outer fascia appears as a thin white line, located below the broad white shadow corresponding to the skin echo at the top of the image. The masseter muscle is visualized as the dark area extending from the fascia to the lateral surface of mandibular ramus at the lower part. During the procedure, real-time dynamic visualization enables the clear differentiation between skin and fascia as muscle alternates from relaxation to contraction. Muscle thickness (A–A) is measured at the thickest point of the masseter muscle between the fascia and the border of the ramus.

With regard to the procedure, participants were seated in an upright position, ensuring a natural head posture. Recordings were taken at the thickest portion of the masseter muscle, typically located near the occlusal plane and at a level midway between the zygomatic arch and the gonial angle. The scanning plane was aligned perpendicular to both the anterior border of the muscle and the surface of the underlying mandibular ramus. Recordings were obtained bilaterally for each subject under minimal occlusal contact (relaxation) and maximal voluntary clenching (contraction). Alternating between relaxed and contracted states allowed better delineation of the superficial muscle borders. All measurements were taken at the thickest region of the masseter muscle and recorded directly on screen to the nearest 0.1 mm (Figure 1). Ultrasonographic measurements were obtained bilaterally in both relaxed and contracted states, with a second set recorded following a five-minute interval. Efforts were made to prevent tissue compression using generous amounts of gel under the probe and minimal pressure. The final value for each state was calculated as the mean of both bilateral measurements. For the purpose of greater reliability of the results, only the measurements of the muscle in contraction were used in this study. When contracted, the muscle maintains its thickness and size, minimizing deformation even under slight pressure during the ultrasonography procedure.

### 2.5. Cephalometric Analysis

Given the evidence indicating an interrelationship between craniofacial morphology and thickness of masseter muscle, whereby brachycephalic skeletal morphology is associated with greater thickness of muscles compared to other vertical profiles [21,22,23,24], lateral cephalograms were assessed to describe the vertical skeletal relationships within the study sample. In addition, the sagittal cephalometric relationships were evaluated using the ANB angle. All lateral cephalograms were digitized and analyzed using the Bergen cephalometric method (Hasund, 1977) [25]. Tracings were generated in a single session by one examiner. To ensure consistency, all cephalograms were standardized for magnification prior to tracing and measurement.

### 2.6. Body Mass Index Assessment

For all subjects in both groups, body weight and height were recorded at T0. BMI was calculated using the standard formula (weight in kilograms divided by height in metres squared; kg/m^2^). Measurements were performed using a calibrated digital scale and stadiometer, with participants in light clothing and no shoes.

### 2.7. Cervical Vertebral Maturation Assessment

Skeletal maturation was evaluated on digital lateral cephalograms using the improved cervical vertebral maturation method suggested by Baccetti et al. [26]. No identifiable patient information was included in the cephalograms and all images were coded for subject matching prior to evaluation. The morphology of the second, third and fourth cervical vertebrae (C2, C3 and C4) was examined, and each subject was classified into one of five cervical vertebral maturation stages (CVMS I to CVMS V), according to the presence and depth of concavities along the inferior vertebral borders and the shape of the vertebral bodies. During the evaluation, particular attention was paid to differentiate true concavities from minor artefacts, such as spikes, to avoid misclassification. When necessary, image contrast and brightness were adjusted to improve visualization of the vertebral borders. CVMS staging was performed on a single lateral cephalogram per subject. All evaluations were performed by one examiner.

### 2.8. Statistical Methods

Statistical analyses were conducted using SPSS, version 29.0 (IBM, Chicago, IL, USA). Statistical significance was set at *p* < 0.05. Parametric tests were performed, since the data were checked and confirmed for normal distribution. Baseline characteristics for the two groups were presented in terms of means and standard deviations and an independent-sample *t*-test was used to compare the baseline characteristics between groups, (age, skeletal characteristics, etc.). Paired-sample *t*-tests were carried out within each group regarding the masseter muscle thickness changes between T0 and T1 time points. Independent-sample *t*-tests were used to assess any potential differences in masseter muscle thickness between the treatment and control group with respect to T0, T1. Finally, multiple regression analysis was performed to examine the association of the masseter muscle thickness changes to several independent variables, including age at T0, initial masseter muscle thickness (MMT0), gender and group allocation (treatment-control) [27].

### 2.9. Error of Method

Ultrasonographic recordings of the masseter muscle thickness under contraction were obtained by a single operator from 20 adult subjects on two different occasions with an approximate one-month interval between the recordings. Random error was calculated using Dahlberg’s formula, namely Se = √(Σd2/2n), where d is the difference between the two recordings of the subject and n the number of double recordings [28], and it was found to be 0.2 mm during contraction, showing consistency of measurements. A paired-sample *t*-test was also performed to assess systematic error [29], with no statistically significant systematic error found at the 5% level, confirming the validity of the measurements.

## 3. Results

### 3.1. Demographics

Thirty-eight subjects were included in the present study, of whom 20 had orthodontic treatment with different types of appliances, while 18 children served as untreated controls.

#### 3.1.1. Orthodontic Treatment Group

The initial mean body mass index (BMI) was 18.45 ± 2.76. According to Angle’s classification, the sample included 11 children with Class I, eight with Class II, and one with Class III malocclusion. One child had an impacted maxillary canine and two children had congenitally missing maxillary lateral incisors. In addition, four children had posterior cross-bite. Regarding vertical dental relationships, 17 subjects had a normal overbite (0–4 mm), one had a deep bite (5 mm), and two had an anterior open bite (>0 mm). The skeletal vertical morphology, assessed by the mandibular plane angle (NSL-ML) and the intermaxillary angle (NL-ML), showed initial mean values of 35.1° ± 4.2° and 25.7° ± 4.1°, respectively. The skeletal sagittal relationships, determined by the ANB angle, demonstrated a mean 4.24° ± 2.01°. According to the cervical vertebral maturation assessment, 10 patients were in the prepubertal, four in the pubertal, and six in the postpubertal stage.

With regard to the treatment modalities, 10 individuals were treated with interceptive appliances (quad helix, lingual arch, transpalatal arch, removable plates) and four patients received functional appliance treatment (activators). The remaining six subjects began treatment with interceptive appliances and subsequently continued for at least 10 months with fixed appliances. Among the treated subjects, four had extractions of primary and/or permanent teeth.

#### 3.1.2. Control Group

The mean BMI of the control group at baseline (T0) was 19.14 ± 4.34. Based on the Angle classification, the group comprised six children with Class I, 11 with Class II, and one with Class III malocclusion. The initial skeletal vertical morphology showed mean NSL-ML and NL-ML angles of 33.4° ± 4.9° and 25.8° ± 4.8°, respectively, and the sagittal skeletal morphology was characterized by a mean ANB angle of 3.57° ± 3.33° (Table 1). Assessment of skeletal maturation indicated that nine children were in the prepubertal stage, two in the pubertal stage, and seven in the postpubertal stage.

At baseline, no statistically significant differences were observed between the groups in age, body mass index (BMI), intermaxillary sagittal relation (ANB), mandibular plane angle (NSL-ML), or vertical intermaxillary angle (NL-ML) (Table 1).

### 3.2. Masseter Muscle Changes

The mean baseline masseter muscle thickness (MMT0) was 11.7 ± 1.4 mm in the treatment group and 11.4 ± 1.3 mm in the control group, with no statistically significant difference observed between the groups (*p* = 0.403). After the approximate one-year follow-up period (14 months average period in the treatment group and 13 months in the control group), the mean masseter muscle thickness (MMT1) was 11.1 ± 1.1 mm and 11.9 ± 1.3 mm in the treatment and control groups, respectively. This difference was statistically significant (*p* = 0.049), with untreated children exhibiting thicker masseter muscles than those undergoing orthodontic treatment (Table 2).

After one year, the orthodontic treatment group showed a significant decrease in masseter muscle thickness (−0.6 ± 0.7 mm; *p* < 0.001), whereas the control group exhibited a significant increase during the same period (+0.5 ± 0.6 mm; *p* < 0.001).

To account for potential confounding factors such as age variation, gender distribution, and initial masseter muscle thickness, a multiple regression analysis was performed. The model included four independent variables, namely, age, gender, baseline masseter muscle thickness, and treatment group, in order to assess their association with the change in masseter muscle thickness over the study period (dependent variable). Based on this analysis, orthodontic treatment was significantly associated with a reduction in masseter muscle thickness with group assignment (treatment vs. control) emerging as the strongest predictor of changes in masseter muscle thickness (β = −1.079; *p* < 0.001). Baseline age and gender had no significant predictive value, whereas initial masseter thickness was a significant factor influencing the changes in the masseter muscle, indicating that subjects with thicker muscles at baseline experienced a greater reduction in thickness following treatment. The model explained 62.7% of the variance (R = 0.792; *p* < 0.001) in the masseter muscle changes (Table 3). The importance of the initial thickness of the masseter muscle in the one-year changes was tested separately in the control and orthodontic patient groups. It was found that the initial thickness of the masseter muscle was significant only in the subjects undergoing orthodontic treatment, with more decrease in masseter muscle thickness in subjects with thicker muscles (*p*< 0.009).

Despite the small number of patients in the different treatment subgroups and the possibility to perform meaningful statistical analyses between changes in masseter muscle thickness and the specific type of orthodontic treatment undertaken, an indicative presentation was attempted. The mean changes (±SD) in the thickness of the masseter muscle in the three subgroups were as follows, all being smaller than those observed in the control group: Patients treated with interceptive appliances presented a change of −0.6 mm (±0.7; *p* < 0.001); those treated with functional appliances showed a change of −0.8 mm (±0.7; *p* < 0.001); and those treated with fixed appliances showed a change of −0.5 (±0.7; *p* < 0.001).

## 4. Discussion

The findings of the present study indicate that masseter muscle thickness is influenced by orthodontic treatment during the growth period. After approximately one year of orthodontic therapy, children in the treated group exhibited an average reduction of 0.6 mm in masseter muscle thickness. Despite the variability in the orthodontic appliances used, this negative effect was consistently observed in the majority of the examined patients. In contrast, children in the untreated control group demonstrated an average increase of 0.5 mm in masseter thickness during the same period, reflecting the physiological muscle growth that naturally occurs in this growth period. Consequently, the orthodontically treated children in our sample exhibited an average muscle atrophy of approximately 1.1 mm compared with the thickness they would have attained through normal growth in the absence of orthodontic treatment. These results suggest that orthodontic interventions may alter the normal growth pattern of the masticatory muscles in growing individuals, leading to a measurable reduction in masseter muscle thickness. Therefore, the null hypothesis can be rejected.

The present findings regarding physiological changes in masseter muscle thickness during growth are in line with previous investigations. Cross-sectional studies of children of comparable ages have reported differences of approximately 0.4–0.5 mm in contracted masseter thickness between groups differing by one year in age [30]. Similarly, longitudinal studies in healthy children have documented average annual increases of 0.5–0.6 mm in masseter muscle thickness [20,31]. Moreover, our findings regarding the decrease in masseter muscle thickness among subjects undergoing orthodontic treatment, are in agreement with the results of Kiliaridis et al. [20], who reported a significant reduction of 0.4 mm in masseter thickness after 13 months of treatment in children using a twin-block appliance. Similarly, a decrease of 0.1 mm in masseter muscle thickness was observed after one year of activator therapy, although this change did not reach statistical significance [31]. Nevertheless, both studies demonstrated that children treated with functional appliances exhibited a reduction in masseter muscle thickness compared with the thickness that would have been expected under normal growth conditions without orthodontic intervention.

Other studies conducted in adult patients treated with clear aligners suggest that clear aligner therapy may induce neuromuscular adaptations characterized by altered recruitment patterns of the masseter and temporalis muscles, accompanied by a concurrent reduction in maximal bite force. These changes appear to persist independent of the presence of the aligners, indicating a central modulation of muscle activity [32]. Similar results have been reported in adult female subjects, where cone-beam computed tomography (CBCT) analysis revealed a significant decrease in masseter muscle dimensions during orthodontic treatment [33].

In the present study, the degree of masseter muscle atrophy observed appeared to be negatively correlated with the initial muscle thickness. Specifically, individuals with thicker masseter muscles at baseline experienced a greater reduction in muscle thickness during treatment. This finding may suggest that masseter muscles are proportionally responsive to the altered functional demands imposed by orthodontic therapy, undergoing a proportional atrophy depending on their initial thickness.

One possible explanation for these findings involves changes in dietary consistency during the initial phases of active orthodontic treatment—a period typically characterized by the patient’s adaptation to an altered oral environment. During this phase, patients often avoid sticky, hard, or fibrous foods, as well as certain nutrient-dense items, in an effort to reduce discomfort and sensitivity [34,35,36]. These dietary modifications lead to a preference for softer foods and altered mastication patterns, reducing both the rate and intensity of chewing activity. This preference for low-resistance foods decreases the mechanical load transmitted to the masticatory muscles. Such reduction in functional demand can induce a detraining effect, reflected in lower muscular stimulation and, consequently, a reduction in masseter muscle thickness.

A further possible explanation for the observed reduction in masseter muscle thickness is that orthodontic treatment alters occlusal contacts, thereby modifying proprioceptive input and reducing muscle activity [37]. Such decreased stimulation can lead to a detraining effect on the masticatory muscles, resulting in mild atrophy and a subsequent reduction in muscle thickness. It is well established that skeletal muscles adapt to functional demands, increasing in size and strength with regular training. This principle also applies to the masticatory muscles. Kiliaridis et al. demonstrated that regular functional training of the masticatory muscles can influence their functional capacity and increase the strength of these muscles [38]. Moreover, studies on individuals with bruxism, regardless of age, indicate that they exhibit greater masseter muscle thickness than average. This is related to excessive muscular activity and continuous exercise [39,40,41]. Therefore, if increased activity and training can lead to hypertrophy, it is reasonable to hypothesize that a detraining effect, due to less stimulation, may lead to a decrease in the thickness of these muscles.

A comparable situation is observed in other skeletal muscles following periods of reduced activity. For instance, immobilization after lower-limb fractures results in muscle mass loss due to disuse and lack of mechanical loading [42]. Likewise, critically ill patients in intensive care units often experience muscle atrophy due to several risk factors, including prolonged inactivity [43]. Nevertheless, the degree of atrophy associated with orthodontic treatment is considerably less pronounced. During orthodontic therapy, masticatory muscle activity may be temporarily diminished due to occlusal instability or discomfort, but the muscles remain functionally engaged in essential activities such as speaking and chewing. Even though dietary modifications during treatment may lower masticatory demands, a baseline level of muscle activity is maintained, thereby preventing extensive atrophy and preserving a substantial proportion of muscle thickness, as observed in the present study.

A limitation of the present study is the small sample size that reduces the possibility of performing thorough subgroup analyses, and the possibility to perform meaningful statistical correlations between changes in masseter muscle thickness and the specific type of orthodontic treatment undertaken, according to the appliances used. The inclusion of different treatment modalities in the sample introduces additional variability, as various appliances may exert distinct functional effects on the masticatory muscles. Future studies may be able to help clarify if different treatment approaches could have a different influence on the masticatory muscles.

Another limitation of this study is that lack of random allocation may introduce selection bias, which is one of the weak points in a cross-sectional study, as is the case in the present one. In favour of our study is the fact that all participants in both groups were drawn from the same clinical setting. Furthermore, no remarkable baseline differences were found among the examined variables between the groups at T0, regarding age, BMI, or skeletal vertical and sagittal relationships. Additionally, the groups showed comparable initial masseter muscle thickness values. This suggests that selection bias, if present, is unlikely to have influenced the baseline muscle characteristics relevant to our outcome measure.

The follow-up period was limited to one year, in order to assure that almost all patients undergoing any kind of orthodontic treatment could be eligible to be included in the patient group. The scope of the present study was firstly to detect possible differences in the masseter muscle thickness after one year, and as it seems that this is indeed the case, to continue with future prospective studies to elucidate if there is further aggravation of the masseter muscle atrophy during orthodontic treatment and if there is post-treatment adaptation, for example, with a catch-up effect after the end of the active orthodontic treatment.

Nevertheless, our retrospective study, despite its limitations in respect to the benefits of a prospective randomized clinical trial, has evoked a situation that was not previously observed in a heterogeneous group of orthodontic patients. It may thus be interesting in this context to use these data to better interpret situations of functional or biological changes during or after orthodontic treatment. Future studies with a prospective design and larger sample size may elucidate further questions on this issue.

## 5. Conclusions

The present study demonstrates that, in our sample, children undergoing orthodontic treatment with different kinds of appliances show a decrease in their masseter muscle thickness one year into treatment, in contrast to the physiologic thickness increase that occurs in children without any orthodontic treatment. These findings suggest that orthodontic interventions could potentially influence the normal growth pattern of the masticatory muscles in growing individuals, leading to a certain degree of muscle atrophy. More specifically, after one year of active orthodontic treatment, an approximate 9% of atrophy of the masseter muscle was observed in treated individuals, compared to the thickness it would have attained in the absence of any orthodontic treatment.

## Figures and Tables

**Table 1:** Baseline characteristics of the treatment and control groups.

Variable (at T0)	Treatment (n = 20)	Control (n = 18)	*p* Value
Age (yr)	10.4 ± 2.1	9.9 ± 2.0	0.422
BMI (kg/m^2^)	18.5 ± 2.8	19.1 ± 4.3	0.573
ANB angle (°)	4.2 ± 2.0	3.6 ± 3.3	0.470
NSL/ML angle (°)	35.1 ± 4.2	33.4 ± 4.9	0.275
NL/ML angle (°)	25.7 ± 4.1	25.8 ± 4.8	0.930

Values are presented as mean ± standard deviation. BMI = body mass index; NSL = nasion-sella line; ML = mandibular line; NL = nasal line; yr = year. Independent-samples *t*-tests were used to compare groups. No statistically significant differences were observed (*p* > 0.05).

**Table 2:** Masseter muscle thickness measurements in millimetres.

	Treatment Group	Control Group	Difference Between the Groups (Control—Treatment Group)		
	Mean ± SD	Mean ± SD	Mean ± SD	95% CI	*p* Value
T0	11.7 ± 1.4	11.4 ± 1.3	−0.3 ± 0.4	−1.241 to 0.50	0.403 (n.s.)
T1	11.1 ± 1.1	11.9 ± 1.3	0.8 ± 0.4	0.003 to 0.57	0.049
T1 − T0	−0.6 ± 0.7	0.5 ± 0.6			
*p* value	<0.001	<0.001			

CI = confidence interval; SD = standard deviation; T0 = initial recording; T1 = final recording; T1-T0 = time interval of approximately one year; n.s. = not statistically significant.

**Table 3:** Multiple regression analysis to test the significance of: (a) control or treatment group, (b) age of subjects at the beginning of the study, (c) gender, and (d) baseline masseter muscle thickness, on the change in masseter muscle thickness after approximately one year.

Variables	Coefficient *b*	Standard Error	Significance (*p* Value)
Group	−1.079	0.171	<0.001
Age T0	−0.015	0.043	0.722 (n.s.)
Gender	0.030	0.181	0.869 (n.s.)
MMT0	−0.178	0.066	0.011

Regression equation: *(Y)* = *b*_0_ + *b*_1_ × GROUP + *b*_2_ × AGE T0 + *b*_3_ × GENDER + *b*_4_ × MMT0; MMT CHANGE = 2.687 − 1.079 × GROUP − 0.015 × AGE T0 + 0.030 × GENDER − 0.178 × MMT0; Dependent variable *(Y)*: Masseter thickness changes (mm) during in 1 year (MMT CHANGE). *b*_0_ = constant, *b*_1,_ *b*_2,_ *b*_3,_ *b*_4_ = regression coefficients. Independent variables: GROUP, AGE T0, GENDER, MMT0; Significance of the model: *R* = 0.792 (correlation coefficient), *R*^2^ = 62.7% (percentage of explained variance), *p* < 0.001. GROUP—control (=0) or treatment group (=1), AGE T0—age of subjects at the beginning of the study, GENDER—male (=0) or female (=1), MMT0—Mean masseter muscle thickness at T0, n.s.—not statistically significant, MMT CHANGE—change in masseter muscle thickness.

## Data Availability

Data and materials supporting the results or analyses presented in the present paper are available upon reasonable request from the corresponding author.
